# Pleiotropy data resource as a primer for investigating co-morbidities/multi-morbidities and their role in disease

**DOI:** 10.1007/s00335-021-09917-w

**Published:** 2021-09-15

**Authors:** Violeta Muñoz-Fuentes, Hamed Haselimashhadi, Luis Santos, Henrik Westerberg, Helen Parkinson, Jeremy Mason

**Affiliations:** 1 European Molecular Biology Laboratory-European Bioinformatics Institute, Wellcome Genome Campus, Hinxton, UK; 2 MRC Harwell Institute, Harwell, OX11 0RD UK

## Abstract

Most current biomedical and protein research focuses only on a small proportion of genes, which results in a lost opportunity to identify new gene-disease associations and explore new opportunities for therapeutic intervention. The International Mouse Phenotyping Consortium (IMPC) focuses on elucidating gene function at scale for poorly characterized and/or under-studied genes. A key component of the IMPC initiative is the implementation of a broad phenotyping pipeline, which is facilitating the discovery of pleiotropy. Characterizing pleiotropy is essential to identify gene-disease associations, and it is of particular importance when elucidating the genetic causes of syndromic disorders. Here we show how the IMPC is effectively uncovering pleiotropy and how the new mouse models and gene function hypotheses generated by the IMPC are increasing our understanding of the mammalian genome, forming the basis of new research and identifying new gene-disease associations.

**Supplementary Information:**

The online version contains supplementary material available at 10.1007/s00335-021-09917-w.

## Introduction

We still do not have a deep understanding of how mammalian genomes function. Most current genetic research focuses only on a small proportion of genes, many of which have already been studied extensively. This leaves a large amount of the genome under-studied, which results in a lost opportunity to identify disease-associated genes and explore new opportunities for therapeutic intervention (Edwards et al. 2011; Oprea et al. 2018; Stoeger et al. 2018).

The International Mouse Phenotyping Consortium (IMPC) is one of a few programmes focusing on obscure or otherwise poorly characterized and/or under-studied genes to uncover novel aspects of gene function. In turn, these newly available mouse models and gene function hypotheses stemming from the IMPC are forming the basis of new research. Notably, the IMPC broad-based phenotyping pipeline is facilitating the discovery of pleiotropy. Pleiotropy can be defined as the effect of a gene or a variant on multiple traits and plays a central role in biology (Stearns 2010; Wang et al. 2010; Paaby and Rockman 2013) with far-reaching implications for evolution, development, canalization, ageing and disease (Wagner and Zhang 2011). Despite pleiotropy’s central importance in biology, empirical datasets have not been available until recently. The analysis of these datasets is contributing to the understanding of pleiotropy, such as its genomic pattern, evolutionary implications, the evaluation of mathematical models and their theoretical predictions and the verification of hypotheses (Wang et al. 2010, Wagner and Zhang 2011, Saltz et al. 2017; Archambeault et al. 2020; Geiler-Samerotte et al. 2020; Shikov et al. 2020), as well as to identify new gene-disease associations.

In this Commentary, we focus on the relevance of pleiotropy for understanding gene function and, in particular, its critical importance to identify gene-disease associations. Here, we define the term trait as the phenotypes that are deviant in the knockout lines produced by the IMPC in comparison to wild type mice (a significant parameter in IMPC terminology), which allow us to link gene to function. We show how pleiotropy is distributed in the IMPC knockout lines and present compelling examples illustrating how this data is instrumental in making disease associations.

## Hypothesis-free, broad phenotyping pipeline enabling systematic analysis of pleiotropy

**Fig. 1:**
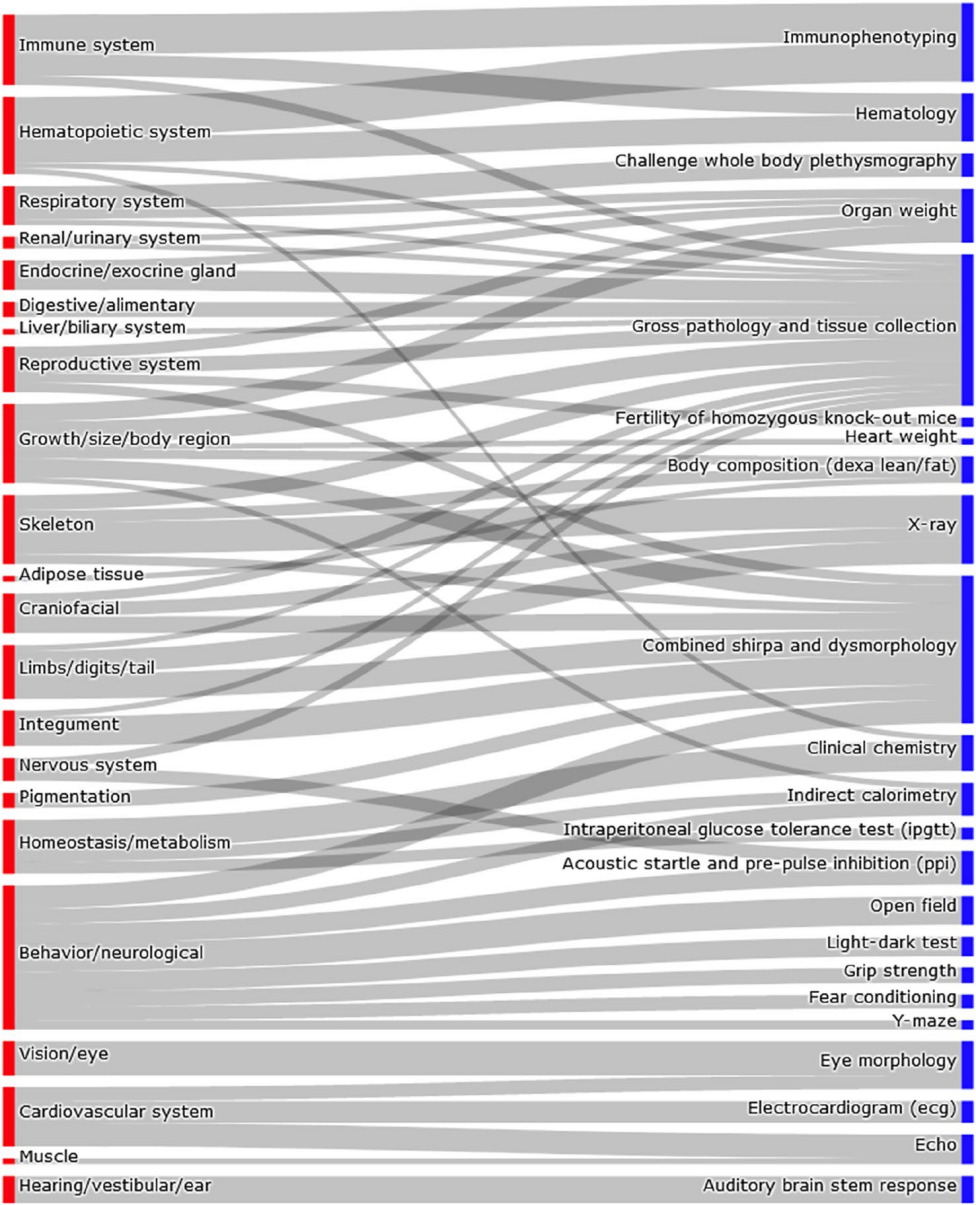
Correspondence between physiological systems and IMPC procedures. The thickness of the lines corresponds to the number of IMPC parameters associated with the physiological system (note that the same parameter can be counted more than once when it is associated with more than one physiological system; see Supp Table 1 for details)

The IMPC is applying hypothesis-free systematic phenotyping screens to characterise single-gene knockout mice. The IMPC phenotyping pipeline is broad, encompassing many physiological systems and parameters (Fig. 1). Phenotypes in the knockout mouse line differing from the controls (wild type) identify the physiological systems that are disrupted when a gene is disabled, thus generating large, multidimensional gene-phenotype associations that are unveiling gene function. Effectively, the null allele can be associated with biochemical, physiological and developmental phenotypic changes. By investigating the impact of a modified gene on multiple organs and physiological systems simultaneously, the IMPC pipeline is well suited to uncover pleiotropy (Brown et al. 2018; Brown and Lad 2019).

The IMPC is ultimately aiming to discover gene-disease associations. Indeed, the phenotypic tests applied by the IMPC have been selected and extended to enable the discovery of phenotypes that will facilitate modelling human disorders (Brown et al. 2018). In disease, pleiotropy manifests as multi-morbidities. In some cases, variants in one gene can lead to apparently unrelated phenotypes (Cerrone et al. 2019). Thus, characterizing pleiotropy is important in the identification of the genetic causes of any disease and, in particular, in the case of syndromic disorders. Some examples using IMPC data are provided below (see section “Associating Genes with Disease” for more detail on this).

In addition to the characterization of adult young mice, the IMPC has two additional pipelines, conducted at the embryonic developmental stage and on aged mice. The assessment of morphology at embryonic stages allows us to characterize early-in-life lethal mutations, with 3D imaging and micro-CT data revealing phenotypes that would have been otherwise missed by gross inspection (Dickinson et al. 2016). Thus, the IMPC pipeline can detect malformations such as spina bifida, abnormal curvature in the spinal column, discoloration, kidney or spleen defects, hypoplastic lungs or thin myocardium, among others (Dickinson et al. 2016). The ageing pipeline investigates phenotypes appearing late in life. The IMPC phenotyping strategy has so far been instrumental in identifying phenotypes for genes of unknown function and provided the first hints for extensive pleiotropy (White et al. 2013; Hrabe de Angelis et al. 2015). It also provides the means to reveal the genetic networks intervening across diverse systems (Brown and Lad 2019).

Here, we investigate the pleiotropic effects of the genes for which the IMPC has produced and phenotyped knockout lines. Generally, the IMPC phenotypes 7 males and 7 females to characterize a mouse line (a cohort of mice deriving from the same gene modification event). An average of 153 parameters were collected per individual mouse. Parameters that show a significant deviation in the mutant cohort from the wildtype mice inform us about the physiological systems that are disrupted when the gene is disabled (Fig. 1). For example, the parameters in the IMPC Echocardiogram procedure (IMPC_ECHO; described in detail here https://www.mousephenotype.org/impress/ProcedureInfo?procID=654) assess the functionality of the heart, thus informing us about the cardiovascular system (Fig. 1). Some IMPC parameters are associated with more than one physiological system (e.g., tibia length is a parameter of the X-ray procedure that is used to assess the limbs as well as the skeleton) and more than one parameter may inform about (sometimes different aspects of) the same physiological system (e.g. the ECHO and ECG electrocardiogram procedures about the cardiovascular system). Supp Table 1 shows a complete list of parameters and the physiological systems they inform.

The IMPC utilises a set of statistical methods (Haselimashhadi et al. 2020) as well as manual curation to determine the deviation of the knockouts from the wildtypes. Significant deviations (at the level of 0.0001) are annotated using Mammalian Phenotype Ontology (MPO) terms (Smith and Eppig 2015). All tests and procedures are described in detail in IMPReSS, the International Mouse Phenotyping Resource of Standardised Screens (https://www.mousephenotype.org/impress). Registering significant results to ontology terms has two main advantages. First, the systematic annotation of well-characterized phenotype descriptions ensures no loss of information and enables us to treat the results informatically (Deans et al. 2015; Thessen et al. 2020). And, secondly, ontologies enable the possibility of systematically implementing cross-species comparisons. To elucidate gene-disease associations, mouse phenotypes expressed using MPO terms are mapped to those of patients whose clinical descriptions are annotated using the Human Phenotype Ontology (HPO; Robinson et al. 2008). More detail on this is provided below.

## Detecting pleiotropy

Mouse lines for a total of 7590 genes (Supp Table 2) have undergone or are currently undergoing phenotyping by the IMPC (DR 14.0, 7 May 2021, www.mousephenotype.org). The IMPC focuses on phenotyping null homozygotes, that is, both alleles have been knocked out or made inactive. Thus, the default strategy is to apply the main IMPC phenotyping pipeline to null homozygotes. However, when the null homozygotes result in a lethal phenotype, the main phenotyping pipeline is applied to the heterozygotes and the null homozygotes are further investigated using the embryo pipeline as described above. Here we report pleiotropy in homozygous and heterozygous mouse lines.

Among the 7590 genes, we distinguish two special cases of genes, those for which the null homozygotes result in a lethal phenotype (lack of homozygous viable individuals) and those genes for which the individuals cannot be associated with any trait (no detectable effect in the knockout line). Essential genes can be argued to be maximally pleiotropic from a developmental or selectional point of view, even if moleculary they only affect one or a few gene’s activities (Paaby and Rockman 2013). In the IMPC data, 24% of the genes present a lethal phenotype (absence of viable homozygotes or complete penetrance of preweaning lethality), while 9% present a variable degree of subviability (incomplete penetrance of preweaning lethality in null homozygotes; it is worth noting that heterozygotes are almost exclusively viable with only two lines presenting a degree of subviability). The IMPC has focused papers dealing with essential genes (Dickinson et al. 2016; Muñoz-Fuentes et al. 2018, Cacheiro et al. 2020), and we refer the interested reader to those publications. In the case of the other special gene class, identifying genes affecting zero traits requires considering a minimum number of phenotypic screens conducted on the mutant line in order to be able to conclude that the effect of the null allele is likely to be negligible (albeit in this particular background and experimental setting). We established this minimum threshold at 13 IMPC procedures. In the IMPC dataset, there are 5146 genes that fulfil this criterion and, out of them, 457 genes have no trait association. Genes with no trait association have been suggested to be an interesting class with undefined pleiotropy (Paaby and Rockman 2013).

**Fig. 2:**
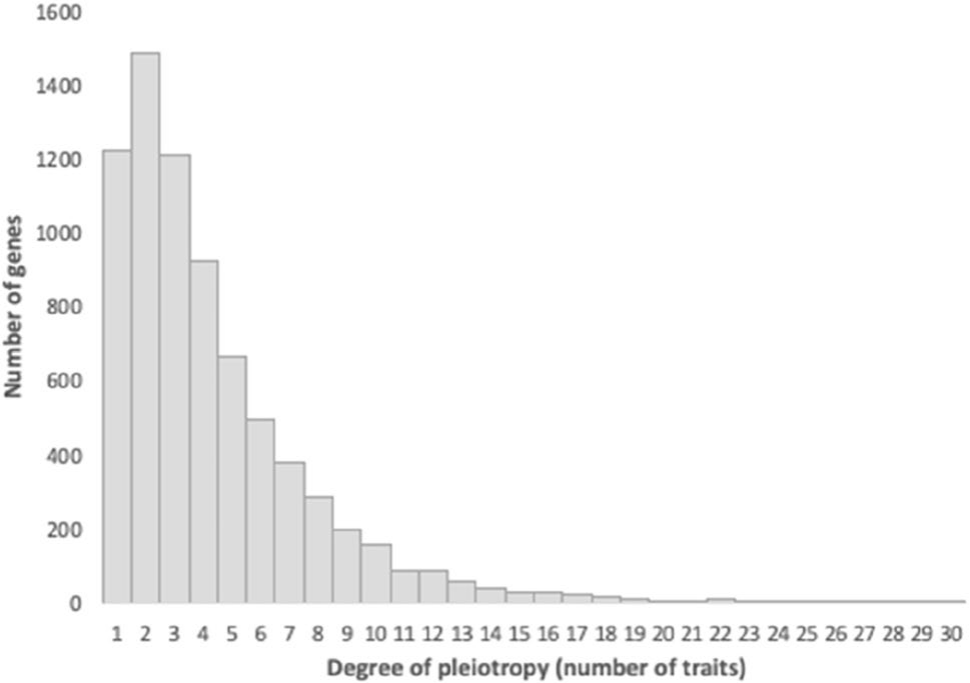
Frequency distribution of the degree of gene pleiotropy showing extensive pleiotropy for 7590 genes studied by the IMPC; 4862 (77%) of the genes affect two or more phenotypes or traits. Degree of pleiotropy here is equivalent to the number of traits

The wealth of IMPC procedures applied to single-gene knockouts for 7590 genes resulted in 1491 genes associated with one trait and 4862 genes (77%) associated with two or more traits. These results indicate that pleiotropy is widespread, but most genes in the genome are not highly pleiotropic and a few affect a great number of traits (Fig. 2).

For the genes affecting two or more traits, 4157 genes showed effects spanning across at least two physiological systems (as defined in the IMPC pipeline, Fig. 1, in turn dictated by the Mammal Phenotype Ontology, Smith and Eppig 2015); it is worth noting that some of these systems are highly related, such as the hematopoietic and the immune systems and certain traits may be mapping to more than one system. Indeed, for the genes affecting one trait, 139 genes had effects that spanned across two or three systems, due to the mapping of those traits to more than one physiological system (Fig. 1).

**Fig. 3:**
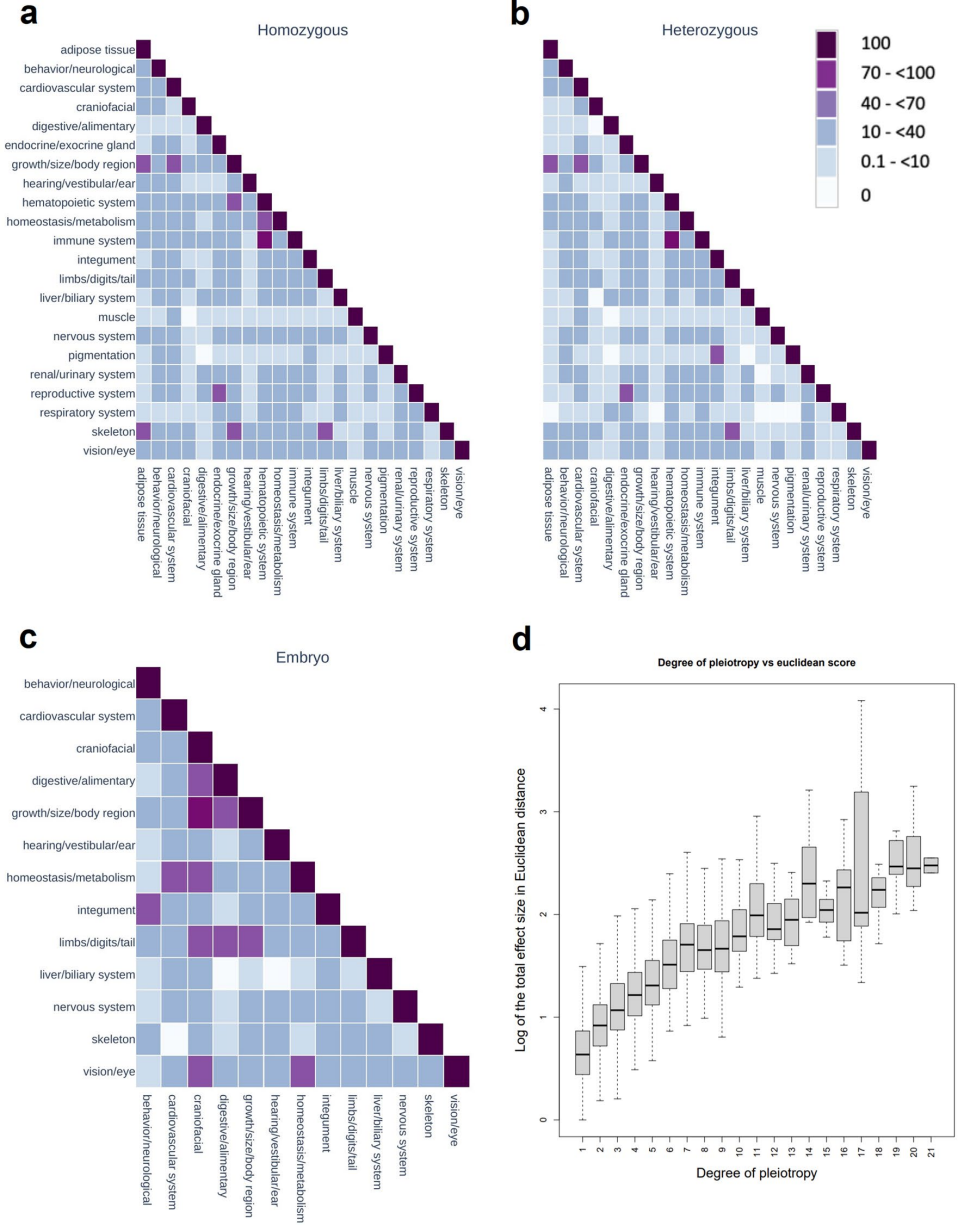
Pleiotropy across physiological systems and in relation to the effect size of the gene on the trait. Co-occurrence matrix of genes by physiological system for viable homozygotes (**a**) and heterozygotes (**b**), assessed with the early adult phenotyping pipeline, and lethal homozygotes (**c**), assessed with the embryo pipeline. Relationship between the total effect size of gene-trait associations in Euclidean distance versus the degreed of pleiotropy (**d**)

We explored this relationship further using a normalized co-occurrence matrix which shows the degree of association of any two physiological systems based on the count of genes that affect traits associated with those two systems. We normalized using the geometric mean to give an idea of the strength of the association based on the underlying data. We show this for early adults (homozygote and heterozygote lines) and embryos (homozygotes not surviving to preweaning stage; Fig. 3a–c). Generally, the homozygotes show higher degree of pleiotropy, a pattern that can be expected as the complete inactivation of the gene normally leads to more abnormal phenotypes than a partial inactivation. Perhaps not so surprisingly, genes affecting the hematopoietic system also affect the immune system (Fig. 3a, b), while other, potentially more interesting relationships may include genes affecting the skeleton also having an effect on adipose tissue, or genes affecting the digestive system not normally having an effect on pigmentation phenotypes (Fig. 3a, b).

These associations require careful consideration of the underlying traits. Evaluating these co-occurrence matrices in the context of the complete list of parameters to associated phenotypic systems (Supp Table 1) is necessary to get an overall picture of how pleiotropy is represented in the IMPC dataset. By design, a single term in the MPO may inherit semantic meaning from multiple parents at each level of the ontology, thus providing the potential for multiple paths from a single highly-specific term to multiple broadly descriptive terms, e.g., a specific term, “abnormal leukocyte physiology,” relates to both “hematopoietic system phenotype” and “immune system phenotype”. For example, many genes associated with the cardiovascular system are also growth/size phenotypes due to annotation with “enlarged heart” and “increased heart weight” phenotypes (Fig. 3a, b), and the overlap of genes associated with craniofacial and digestive systems in embryos is driven by the identification of a “cleft palate” phenotype (Fig. 3c). In contrast, the association between the skeleton and adipose tissue (Fig. 3a) is not driven by phenotypes that annotate to both systems (there are none, Supp Table 1; e.g. *Prdm14, Ube4a, Arl10, Dnd1*). While phenotypes of the respiratory systems, muscles or digestive system have few associations with phenotypes in other systems, this could be a consequence of the way we measure e.g., number of traits investigated to obtain a full characterization of that system (Supp Table 1). Thus, further investigation is needed in this space.

A widely cited relationship has been previously described in which a larger per-trait effect size is observed for genes affecting more traits, which has implications for our understanding of the evolvability and the adaptation of organisms (Wagner et al. 2008; Wang et al. 2010). We also investigated this relationship between the degree of pleiotropy and the effect size for each gene (Fig. 3d). To do this, we used a subset of the genes for which we had continuous measurements (Supp Table 3) to calculate the Euclidean distance from the individual gene-trait associations. The effect size is calculated using the Cohen method (Haselimashhadi et al. 2020). Our results based on the extensive IMPC data set are consistent with previous observations.

## Associating genes with disease: mouse strains that model the patients’ phenotypes

Detecting co-morbidities certainly plays a role in the association of genes with disease in humans and the IMPC broad-phenotyping pipeline attempts to capture the collection of phenotypes that may be manifested when a gene is disabled. Key to this endeavour is matching the comprehensive collection of mouse phenotypes associated with a gene with the patients’ phenotypes. To identify models of human diseases, including rare diseases, the IMPC applies a translational pipeline which uses the PhenoDigm algorithm, developed by the Monarch Initiative (Smedley et al. 2013), the human phenotype ontology (HPO) annotations associated with a disease, maintained by the Monarch Initiative (Köhler et al. 2021; Mungall et al. 2017) and the Mammalian Phenotype Ontology (MPO) annotations from both the Mouse Genome Database (Bult et al. 2019) and the IMPC. A score, between 0 and 100, provides a quantitative measure of the phenotypic similarity between a particular mouse strain and the clinical description of a disease. The results of the pipeline are integrated in the IMPC website. Mouse strains, by modelling the patients' phenotypes, help to establish gene-disease associations.

**Fig. 4:**
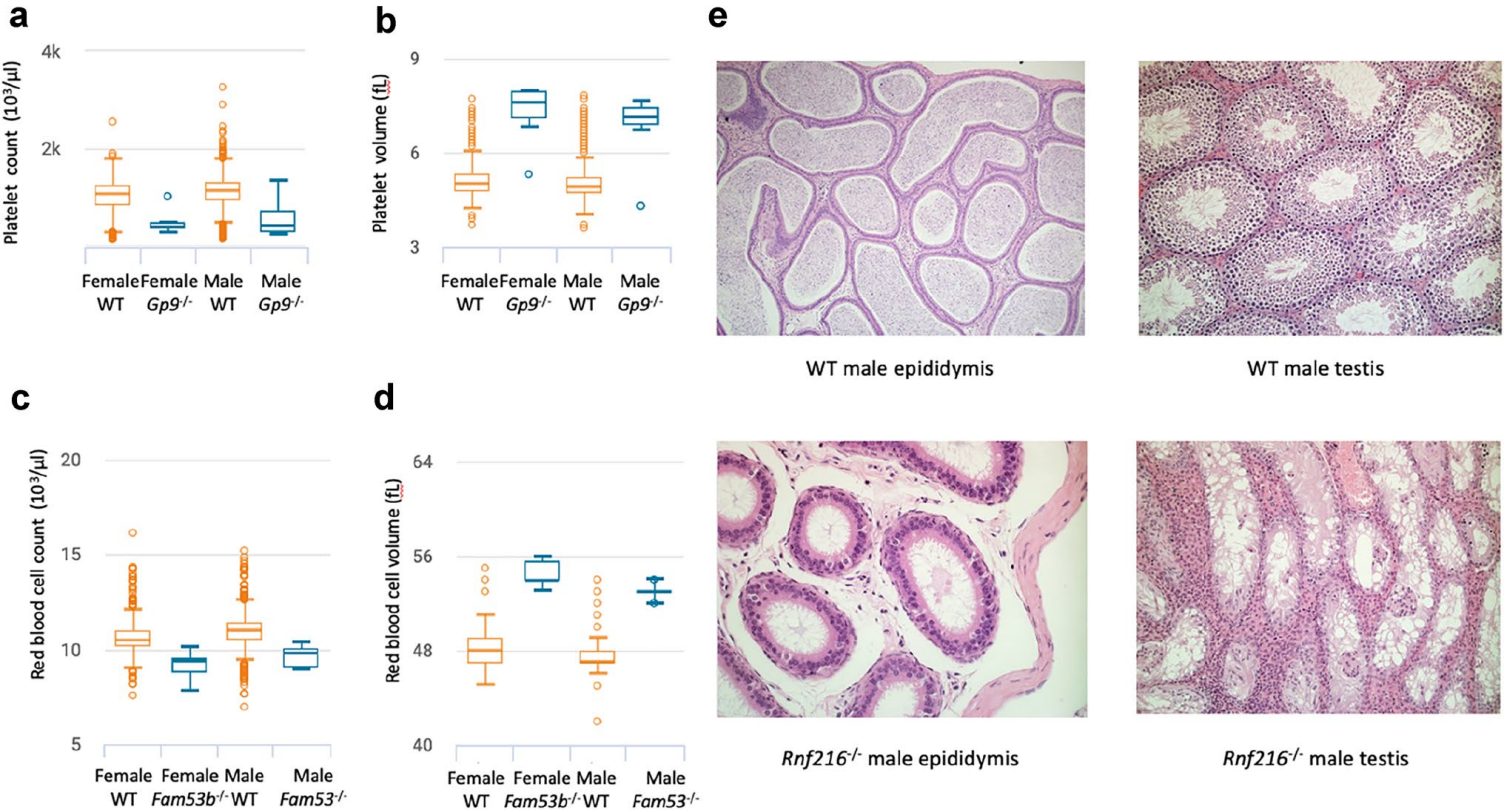
Selected traits of mouse models for Mendelian disease identified using the IMPC translational pipeline. *Gp9*^*tm1.1(KOMP)Vlcg*^ homozygotes have decreased platelet number (**a**) and increased platelet volume (**b**). *Fam53b*^*tm1b(EUCOMM)Hmgu*^ homozygotes have decreased red blood cell number (**c**) and increased red blood cell volume (**d**). *Rnf216*^*tm1b(EUCOMM)Wtsi*^ homozygotes are infertile; histopathology images show epididymal aspermia and testicular degeneration and atrophy and lack of spermatogenesis in the mutants, while the wild types are unaffected (**e**)

This strategy was exemplified in a study focusing on the phenotypes of IMPC knockouts for 3328 genes (Meehan et al. 2017). Some examples from this study included the null homozygotes for *Gp9,* which recapitulated key features of the Bernard-Soulier syndrome, a bleeding disorder, *or Rnf216* homozygous-null mice which, characterized by hypogonadism and cerebellar ataxia, were associated with the Gordon-Holmes syndrome; likewise, *Fam53b* was proposed as a new model for anemia (Fig. 4). In another study, a gene essentiality classification using IMPC (mouse) and human screens was presented as a criterion to prioritise gene candidates for developmental disorders (Cacheiro et al. 2020). The authors presented evidence for the association of *VPS4A* and *TMEM63B* with two unsolved cases associated with intellectual disabilities in humans. The observed phenotypes were relevant for establishing the link between the clinical manifestation of the disease and these genes. The mouse knockouts of the orthologous *Vps4a* gene presented lens opacity and brain abnormalities, and the mouse knockouts of the orthologous *Tmem63b* gene abnormal behaviour, hyperactivity and limb-grasping phenotypes, all consistent with the human patients’ phenotypes.

## Conclusion

The IMPC, with its broad-phenotyping pipeline, is building a pleiotropy-data resource. Data deriving from mouse models suggests that pleiotropy is potentially more widespread than initially anticipated, and that the majority of genes affect only a few traits, and a few affect many traits. We show that the frequency distribution of pleiotropy or its relationship with gene effect sizes based on the wealth of data collected by the IMPC is consistent with previous findings, based on data from mice and other organisms (Wagner et al. 2008; Albert et al. 2008; Wang et al. 2010; White et al. 2013; Hrabe de Angelis et al. 2015; Cerrone et al. 2019). Thus, IMPC data is well suited to contribute to the understanding of the impact of pleiotropy on adaptive evolution and the evolvability of complex organisms, the understanding of the genotype–phenotype maps and the validation of mathematical models and their predictions (Wagner et al. 2008, Wang et al. 2010, Wagner and Zhang 2011).

Notably, the IMPC constitutes a data resource for investigating co-expressed abnormal phenotypes, including those of poorly characterized genes, which makes this functional catalogue indispensable in detecting co-morbidities and identifying genes associated with disease. By combining these data with other resources, such as the Mouse Genome Informatics (MGI) database (https://www.informatics.jax.org) or human essentiality screens, as shown above, the power to identify genes associated with disease can be augmented. Furthemore, the embryo pipeline allows investigation of developmental malformations early in life, which is indispensable in order to understand the effects of lethal genes. Finally, the IMPC, with its specific pipeline applied to aged mice, will facilitate the study of the link between gene and phenotypes appearing later in life, and the implications for disease associations.

Such a resource does not come without limitations. Because of its high-throughput nature, the IMPC pipeline focuses on phenotyping the minimum number of animals necessary to detect strong phenotypic effects (Hrabe de Angelis et al. 2015) and characterizes only protein coding single-gene knockout effects. The phenotypic screens applied by the IMPC have been selected to facilitate modelling human disorders; however, some key biological functions are not fully covered or not investigated (e.g. respiration, inflammation), in some cases only in centre-specific pipelines or pilot studies (e.g. immunophenotyping, https://www.mousephenotype.org/impress/PipelineInfo?id=15; pain pilot study, Wotton et al. 2020; https://www.mousephenotype.org/understand/data-collections/pain/). The goal of the IMPC is to ascribe altered physiological function in the presence of a null mutation, hence additional challenges are intentionally minimised to increase the signal of the mutated genotype. Detecting pleiotropy in the context of gene by environmental interaction would be highly insightful, but falls outside the scope of the IMPC. By working together with the medical and scientific communities this resource may be expanded in the future to produce and characterize more complex genetic modifications of potentially clinical relevance.

The IMPC, through its website www.mousephenotype.org, facilitates access to data and mouse models as they become available (3–4 data releases a year). The IMPC portal not only displays phenotype data and available mouse lines, but also provides access to sophisticated tools to visualize phenotypes encompassing quantitative, categorical and image data. IMPC mice, produced using either embryonic-stem-cell or CRISPR-Cas9 technology, are available for future research. Thus, the IMPC paves the way to increased knowledge about under-studied or poorly characterized genes, as well as to new experiments aiming at increasing our understanding of gene function, disease mechanisms or new therapies.

The IMPC, through its website www.mousephenotype.org, facilitates access to data and mouse models as they become available (3–4 data releases a year). The IMPC portal not only displays phenotype data and available mouse lines, but also provides access to sophisticated tools to visualize phenotypes encompassing quantitative, categorical and image data. IMPC mice, produced using either embryonic-stem-cell or CRISPR-Cas9 technology, are available for future research. Thus, the IMPC paves the way to increased knowledge about under-studied or poorly characterized genes, as well as to new experiments aiming at increasing our understanding of gene function, disease mechanisms or new therapies.

## Supplementary Information

Below is the link to the electronic supplementary material.

Supplementary file1 (XLSX 64 kb) Collection of parameters collected by the IMPC, organised by procedures, and the physiological systems they inform

Supplementary file2 (XLSX 187 kb) List of genes and IMPC alleles included in this study

Supplementary file3 (XLSX 445 kb) List of genes with continuous measurements used to show the relationship between the total effect size of gene-trait associations including Euclidean score versus the degree of pleiotropy used to create Fig. 3d
